# Sr(ii) extraction by crown ether in HFC: entropy driven mechanism through H_2_PFTOUD†

**DOI:** 10.1039/d2ra04411k

**Published:** 2022-09-21

**Authors:** Kenji Shirasaki, Mitsuie Nagai, Masahiko Nakase, Chihiro Tabata, Ayaki Sunaga, Tsuyoshi Yaita, Tomoo Yamamura

**Affiliations:** Institute for Materials Research, Tohoku University Sendai Miyagi 980-8577 Japan kenji.shirasaki.e4@tohoku.ac.jp; Fukushima Reconstruction and Revitalization Unit, Institute of Innovative Research, Tokyo Institute of Technology Tokyo 152-8550 Japan; Institute for Integrated Radiation and Nuclear Science, Kyoto University Kumatori Osaka 590-0494 Japan yamamura.tomoo.2e@kyoto-u.ac.jp; Materials Sciences Research Center, Japan Atomic Energy Agency Tokai Ibaragi 319-1195 Japan; Materials Sciences Research Center, Japan Atomic Energy Agency Kouto Hyogo 679-5148 Japan; Department of Physics, Graduate School of Science, Kyoto University Kyoto 606-8502 Japan

## Abstract

The solvent extraction of Sr(ii) was carried out using dicyclohexano-18-crown-6 (DCH18C6) and two HFC mixed solvents MS1 and MS2, where MS1 was composed of 30/60 (w/w)% *trans*-1,2-dichloroethylene/HFC-43 (HFC-43: 1,1,1,2,2,3,4,5,5,5-decafluoropentane) and MS2 was 5/95 (w/w)% heptane/HFC-43. Nitric acid and perfuruoro-3-6-9-trioxaundecane-1,11-dioic acid (H_2_PFTOUD) were used to study the effect of acid on the extraction. The maximum distribution ratio of Sr(ii) (*D*_Sr_) observed for H_2_PFTOUD conditions was ∼180, and >10 times larger than aqueous nitric acid conditions. The *D*_Sr_ value was influenced by concentrations of the DCH18C6, Sr(ii), and acid, and by temperature. The composition of extracted complexes was estimated using slope analysis as an Sr(ii)–anion–DCH18C6 ratio of ∼1 : 2 : 1. From the extended X-ray absorption fine structure (EXAFS) measurements of Sr(ii) in the aqueous and organic phases, it is inferred that regardless of the acid used, DCH18C6 coordinates to the first coordination sphere of the Sr(ii) extracted complexes and Sr(ii) is hydrated (complexation with H_2_PFTOUD cannot be distinguished) in the aqueous phase. Thermodynamic data were significantly changed by choice of acid, *i.e.*, both enthalpy and entropy values were negative for nitric acid conditions, on the other hand, entropy values were large and positive for H_2_PFTOUD conditions. These results have demonstrated that the combination of HFC solvent and crown ether is applicable for metal extraction.

## Abstract 2

The solvent extraction of Sr(ii) was carried out using DCH18C6, and two HFC mixed solvents composed of organic solvents and HFC-43 (HFC-43: 1,1,1,2,2,3,4,5,5,5-decafluoropentane), and two acids (nitric acid and H_2_PFTOUD).

## Graphical abstract

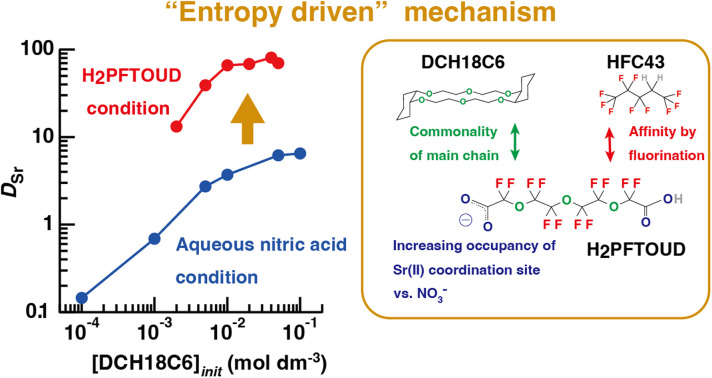

## Introduction

**Fig. 1:**
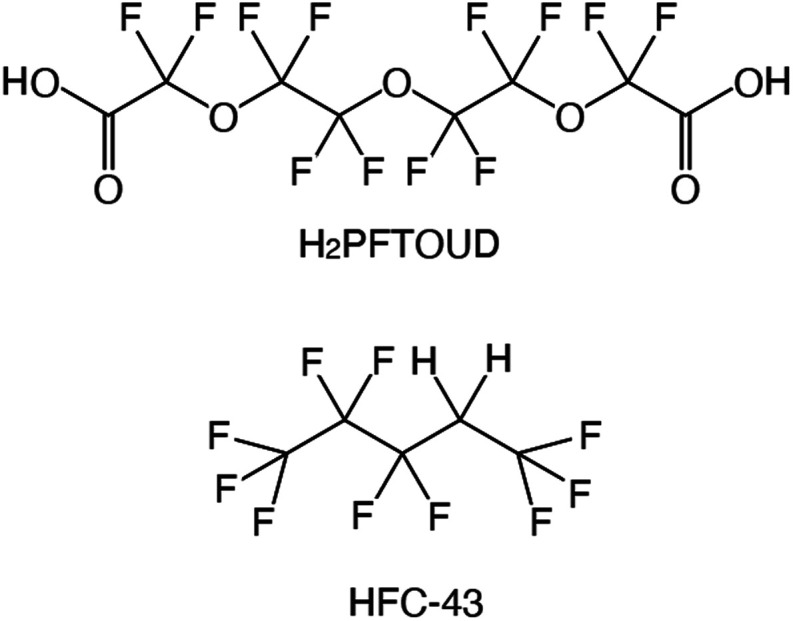
Chemical structures and abbreviations for fluorinated acid and hydrofluorocarbon.

Fluorinated solvents have been of continuous interest in the area of separation and purification technology because of their unique properties such as good extractability, extremely low solubility in water (generally lower than conventional organic solvents), and chemical and radioactive stability. In the UNEX process, phenyltrifluoromethyl sulfone (FS-13) is used with chlorinated cobalt dicarbolide, substituted polyethylene glycol, and carbamoyl phosphine oxide to separate Cs(i), Sr(ii), actinides, and lanthanides from high-level waste (HLW).^1,2^ Some transition metal ions such as Fe(iii), Co(ii), Ni(ii), and Cu(ii) were extracted from water into perfluorohexane (FC-72) and perfluorooctane (FC-3255) with 0.01 mol dm^−3^ (hereafter, abbreviated as “M”) of 1,1,1,5,5,6,6,6-octafluoro-2,4-hexanedione when the pH in the aqueous phase was 1.7–4.8.^3^ The extracted transition metal ions were readily stripped using 1 M nitric acid. In the case of lanthanides, the trend of extraction from water into C_4_F_9_OC_2_H_5_ (HFE-7200) with 4,4,5,5,6,6,7,7,8,8, 9,9,9-tridecafluoro-1,(2-thienyl)-1,3-nonanedione was studied by Nakamura *et al.*^4^ They reported that the extractability of five heavy lanthanide ions such as Lu(iii), Yb(iii), Tm(iii), Er(iii), and Ho(iii) was comparable to that of conventional organic solvent-based extraction systems. We also proposed the use of 1,1,1,2,2,3,4,5,5,5-decafluoropentane (HFC-43, Fig. 1), as a diluent.^5^ Recently, we reported the extraction behaviour of trivalent lanthanide ions (La, Ce, Pr, Nd, Sm, Eu, Gd, Dy, Ho, Er, Tm, Yb, and Lu) and U(vi) from nitric acid medium to HFC-43 using octylphenyl(*N*,*N*-diisobutylcarbamoylmethyl)phos-phine oxide (CMPO).^6^ CMPO showed good solubility in HFC-43 without third phase formation at a concentration of 0.4 M and nitric acid concentration of <4 M.

Functionalized fluorochemicals have surfactant properties and are selectively adsorbed at the interface. In fact, fluorinated surfactants, such as perfluoroalkyl substances (PFAS), constitute an important class of fluorinated compounds utilized in fire extinguishers, herbicide and insecticide formulations, cosmetics, greases and lubricants, paints, polishes, and adhesives.^7^ However, PFAS, especially perfluorooctanoic acid, perfluorooctane-sulfonic acid, and long-chain perfluoroalkyl carboxyl acids (carbon chain length longer than 9), have garnered attention as highly persistent^8^ and toxic^9–11^ environmental contaminants. The industrial sector has shifted toward using alternatives because of the voluntary phase-out of long-chain PFAS by manufacturers. Perfuruoro-3-6-9-trioxaundecane-1,11-dioic acid (H_2_PFTOUD, Fig. 1) is a perfluorinated tetraethylene glycol derivative with oleophobic and hydrophobic chain; its terminal functional groups (*i.e.*, carboxylic acids) impart hydrophilic and polymerization abilities. Recently, Zhu *et al.* developed a method for preparing sodium and potassium salts of H_2_PFTOUD, which is a novel perfluoroalkyl compound with weak bioaccumulation ability.^12^ Moreover, several examples of the use of H_2_PFTOUD can be seen in patents, including as a functional coating on the surface of a semi-permeable membrane composite,^13–15^ a component in treatment fluids used in subterranean formation operations,^16^ and a component in modified thermoplastic elastomers for increased compatibility with supercritical fluids.^17^ However, there is no applicable information on solvent extraction using H_2_PFTOUD, despite its interfacial properties, which are suitable for application in separation and concentration technology.^18,19^

In the field of solvent extraction, strontium is one of the elements of interest.^20^ Because the removal of ^90^Sr (*t*_1/2_ = 28.9 years), contained in HLW produced from nuclear reactors, has been studied to better manage HLW's risk with regard to medium-term hazards.^21^ Crown ethers, especially dicyclohexano-18-crown-6 (DCH18C6) and di-*tert*-butyl cyclohexano-18-crown-6 (DtBuCH18C6), are suitable for the extraction of Sr(ii). Thus, extensive extractions using crown ethers have been developed from the perspective of the treatment and disposal of HLW.^22,23^ The crown ether strontium extraction (CESE) process has adopted DCH18C6 with 1-octanol^24^ and the strontium extraction (SREX) process uses DtBuCH18C6 with carbon tetrachloride as the organic phase.^25^ The diluent effect in Sr(ii) extraction has also been investigated using DCH18C6 (ref. 26) and DtBuCH18C6.^27^ Other than using single extractant, a combination of different extractants is useful for Sr(ii) extraction, *e.g.*, both 18C6 derivatives with didodecylnaphthalene sulfonic acid,^28^ versatic acid,^28,29^ cobalt dicarbolide,^30–32^ di-*n*-octylphosphoric acid,^33,34^ and di(2-ethylhexyl)alkylenediphosphonic acid^35,36^ are used for synergistic extraction. A better understanding of a given extraction process leads to optimize the extraction system. To survey the extraction process, extended X-ray absorption fine structure (EXAFS) as well as distribution analysis can provide useful information. In fact, the interactions of Sr(ii) with *cis-syn-cis* DCH18C6 isomer, and counter ions (NO_3_^−^, Cl^−^ or SO_4_^2−^) were explored using EXAFS measurements both in the room-temperature ionic liquid and 1-octanol by Dietz *et al.*^37^ Their group also reported the EXAFS of Sr(NO_3_)_2_(DtBuCH18C6) on solid support.^38^ Also, thermodynamic data provide information on species formation and ion transfer. Negative enthalpy and entropy values obtained from the Sr(ii) extraction by both 18C6 derivatives into 1-octanol or 1-butanol/1-octanol mixture from 4 M nitric acid, suggested formation of ion-association type species in the extraction process.^39^

Herein, the combination of a fluorinated solvent and crown ether for Sr(ii) solvent extraction was studied. To overcome the low solubility of DCH18C6 in fluorinated solvents, two HFC mixed solvents with HFC-43 used as the base, 30/60 (w/w)% *trans*-1,2-dichloroethylene/HFC-43 and 5/95 (w/w)% heptane/HFC-43, were examined. As a comparative study of the effect of acid, two types of acids (conventional nitric acid and H_2_PFTOUD with fluorophilic and hydrophilic properties) were used (abbreviating the former as ‘aqueous nitric acid conditions’ and the latter as ‘H_2_PFTOUD conditions’, respectively). Enthalpy and entropy data were also obtained in both conditions. In the slope analysis, the extraction equilibrium and the composition of the extracted species were evaluated by the simultaneous equations. The equations containing two undetermined parameters (the conditional extraction equilibrium constant *K*′_ex_ and the coordination number *n* of DCH18C6 on the Sr(ii) extracted species) were solved by applying least square approximation to the calculated distribution ratio and experimental values. Extended X-ray absorption fine structure (EXAFS) measurements were conducted to provide insight into the complexation of Sr(ii) on solvent extraction in both organic and aqueous phases.

## Experimental section

### Materials

Strontium nitrate and DCH18C6 were purchased from FUJIFILM Wako Pure Chemical Co., Ltd, Japan. H_2_PFTOUD was purchased from Matrix Scientific, USA. HFC mixed solvents, 30/60 (w/w)% *trans*-1,2-dichloroethylene/HFC-43 (MS1) and 5/95 (w/w)% heptane/HFC-43 (MS2) were supplied by Chemours-Mitsui Fluoroproducts Co., Ltd, Japan. Other chemicals were purchased from FUJIFILM Wako Pure Chemical Co. and used without further purification. Deionized water (18 MΩ cm, Academic A10 model, Milli-Q, USA) was used in all experiments.

### Extraction of HNO_3_ and H_2_PFTOUD

The acid extractions by DCH18C6 in HFC mixed solvents and the solvent itself were evaluated by contact with the same volume of aqueous acid solution. The two phases were allowed to stand for 12 h after mixing and then carefully separated. The acid concentrations of the aqueous phase were determined using acid–base titration (COM-300A, Hitachi, Ltd). For nitric acid extraction, the organic phase was washed 5 times with an equivalent volume of water to remove all of the extracted acids. The acid concentration of the total washings was determined using UV spectroscopy (UV-3100PC, Shimadzu Co.) because absorption of UV light by nitrate ions is in accordance with the Beer–Lambert law of absorption;^40^ the detection limit was found to be 0.005 mM (Fig. S1†). The concentration of H_2_PFTOUD in the organic phase was estimated from the concentration difference of the aqueous phase before and after extraction.

### Sr(ii) extraction

Two-phase samples were prepared by mixing aqueous and organic phases with equal volumes of 2 mL in 6 mL stoppered tubes. Typical initial concentration of Sr(ii) in the aqueous phase was 0.1 mM. Under aqueous nitric acid conditions, the initial acid concentration was 2 M, except for the experiments studying the acid concentration dependency. The organic phases were prepared by dissolving DCH18C6 in each HFC mixed solvent (MS1 and MS2). H_2_PFTOUD was initially loaded into the organic phases, except under aqueous nitric acid conditions. Typical extractions were conducted using a mixing block (Bioer Technology Co., Ltd), in which the temperature (25 ± 0.5 °C with the exception of the experiments studying the temperature dependency) and vortex mixing velocity (1000 rpm) were controlled. All extractions were conducted with a shaking time of 5 min based on the evaluation of time to reach equilibrium (Fig. S2†). After extraction, the two phases were rigorously separated following an aliquot of the aqueous phase sampled to measure the concentration of strontium using inductively coupled plasma atomic emission spectrometry (ICPS-7500, Shimadzu Co.) or inductively coupled plasma mass spectrometry (Agilent 8900, Agilent Technologies). The distribution ratio (*D*_Sr_) was calculated using eqn (1):1
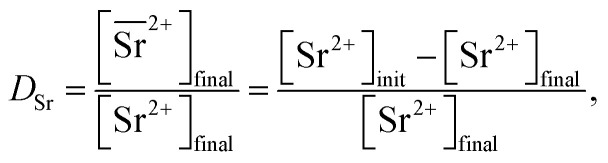
where the subscripts init and final are the initial and final concentrations during extraction, respectively. In addition, the overbar indicates the corresponding species in the organic phase. The data was fitted with an extraction model based on mass balances and equilibrium equations using the EQUATRAN software package (Omega Simulation Co., Ltd, Japan).^41^

### EXAFS measurements and analysis

EXAFS measurements were conducted at the Sr K-edge on the beamline BL-27B of the KEK Photon Factory, in which the X-ray absorption spectra were collected in fluorescence mode. The incident X-rays were monochromatized using Si(111) crystals. For the measurements, the solutions packed in the sample tubes were placed in the X-ray pathway. The absorption data were analyzed using ATHENA software package.^42^

### γ-Ray irradiation

γ-Ray irradiation of the samples was carried out at room temperature at the Takasaki Advanced Radiation Research Institute, Japan. The radiation dose applied to the samples was varied from 0.4 to 25 kGy. After irradiation, extraction experiments were performed using the same procedure described above.

## Results and discussion

### HNO_3_ and H_2_PFTOUD partitioning

Several previous studies have demonstrated that acids are partitioned into the organic phase by the extractant and the organic solvent itself. In the case of DCH18C6, it has been reported that one molecule of DCH18C6 can extract one or two HNO_3_ molecules in a conventional organic solvent.^43^ In contrast, there is no useful information regarding H_2_PFTOUD extraction. In this study, the extraction of HNO_3_ and H_2_PFTOUD by DCH18C6 in HFC mixed solvents was studied for the first time.

**Fig. 2:**
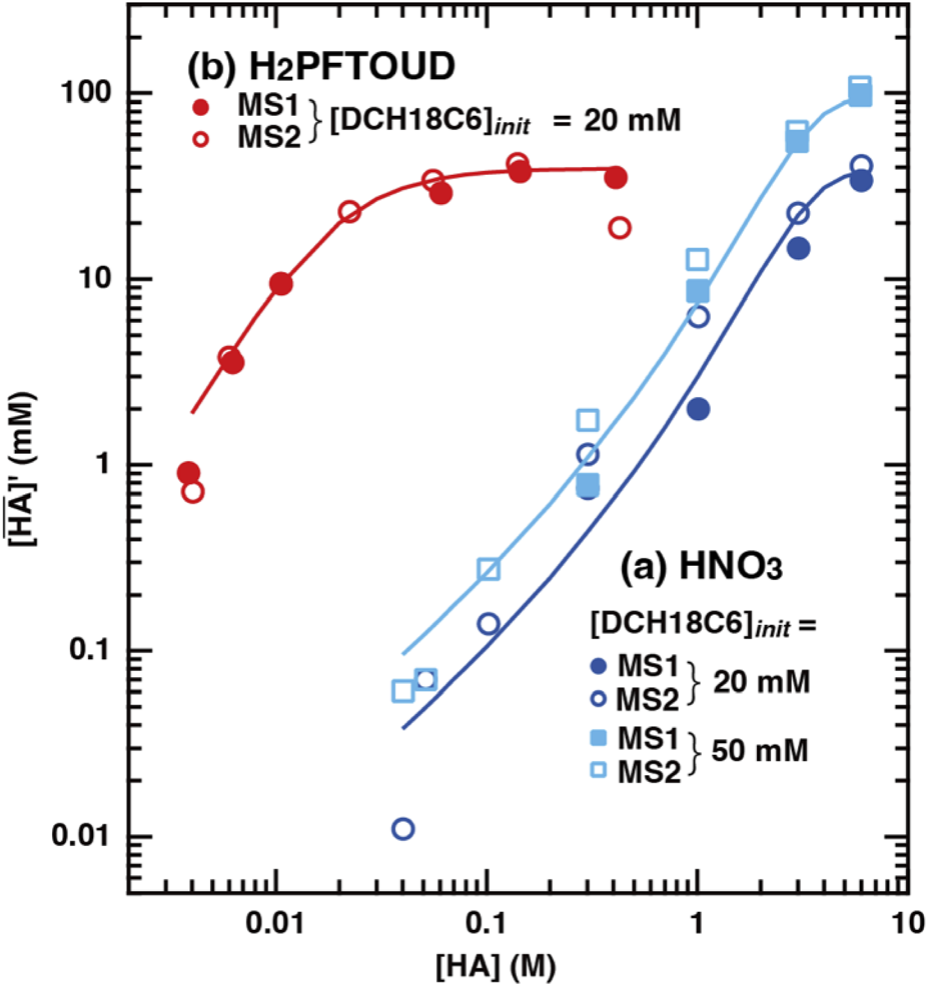
Extraction of (a) nitric acid and (b) H_2_PFTOUD using DCH18C6 in HFC mixed solvents. The fitting curves were assumed as the formation of 1 : 1 and 1 : 2 complexes with DCH18C6 and HA (HA = HNO_3_ (blue and light blue) and H_2_PFTOUD (red)).

Fig. 2(a) shows the corrected concentration of nitric acid in the organic phase (
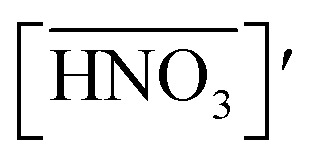
) as a function of the aqueous nitric acid concentration ([HNO_3_]), in which the former has been corrected for the extraction of the acid by the HFC mixed solvent itself. Therefore, 
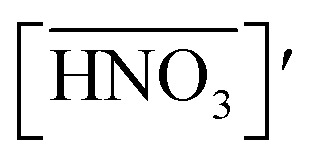
 represents only the acid associated with DCH18C6. The acid concentration extracted by DCH18C6 in the organic phase increased upon increasing the initial concentration in the aqueous phase. The extractability of nitric acid by DCH18C6 was essentially the same between MS1 and MS2 and the amount of extracted acid increased in 0.05 M of DCH18C6 when compared with that in 0.02 M of DCH18C6.

Thus, we attempted to evaluate the behavior of HNO_3_ molecules extracted by DCH18C6 in the organic phase using the extraction constant. Assuming that DCH18C6 (abbreviated as ‘CE’ in following equations) extracts *i*HA molecules (HA = HNO_3_, *i* = 1, 2), the equation is described as follows:2
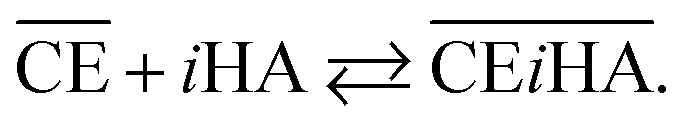


Simultaneously, the extraction constant is expressed as follows:3
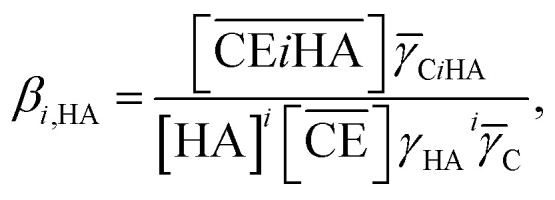
where *γ̄*_C_ and *γ̄*_CiHA_ represent the organic phase activity coefficients of DCH18C6 and its 1 : *i* complex with nitric acid and *γ*_HA_ is the activity coefficient of nitric acid in the aqueous phase.^32^ The organic phase activity constants vary as a function of the nitric acid concentration in the aqueous phase and the DCH18C6 concentration in the organic phase. However, the lack of information available on these activity coefficients makes it necessary to assume that the ratios are constant and can be incorporated into the extraction constant, which can be rewritten as follows:4
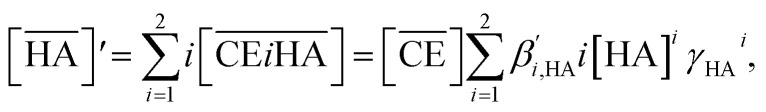
where the prime mark indicates the conditional extraction constant. The mass balance of nitric acid in the organic phase is given by eqn (5) and (6) (if the volumes of the aqueous and organic phases are the same).5
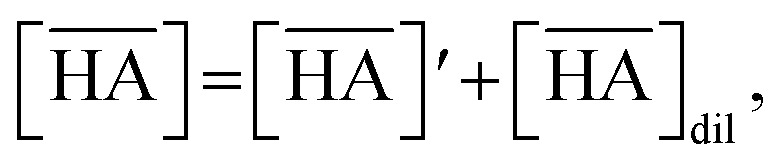
6
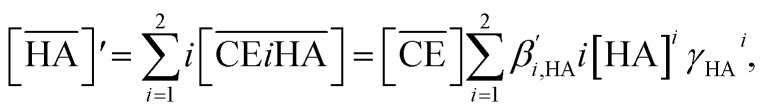
where 
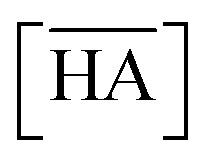
 denotes the concentration of nitric acid in the organic phase, which contains the acid partitioned by the diluent itself (
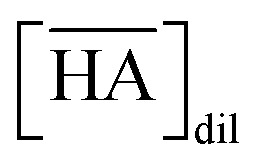
). The results obtained for acid partitioning by the HFC mixed solvent showed an extremely limited ability to extract nitric acid (Table 1). Thus, the partitioning is negligible during acid extraction by DCH18C6, especially for acid concentrations <1 M. The total mass balance of nitric acid in both the organic and aqueous phases can be expressed using the initial aqueous acid concentration ([HA]_init_) as follows:7
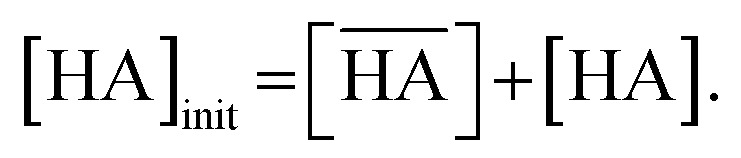


**Table 1:** The partitioning of HNO_3_ into organic phase by HFC mixed solvents

[HNO_3_]_init_ (M)	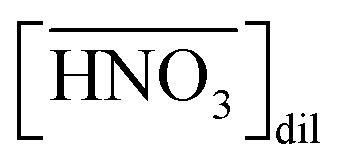 (mM)	
	MS1	MS2
1.0	<0.025	<0.025
3.0	0.50	0.31
6.0	6.78	1.63

Simultaneously, the mass balance of DCH18C6 in the organic phase and the total mass balance for DCH18C6 in both the organic and aqueous phases can be expressed as follows:8
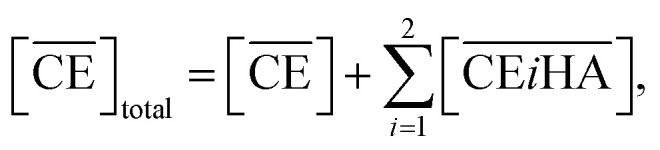
9
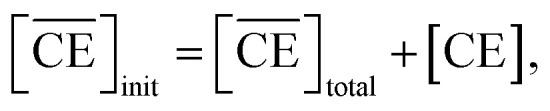
where 
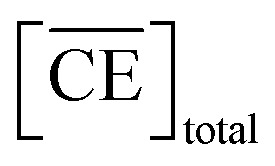
 and 
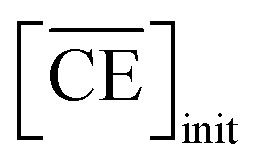
 denote the total and initial DCH18C6 concentrations in the organic phase, respectively. The DCH18C6 distribution ratio (*D*) was expressed as follows:10
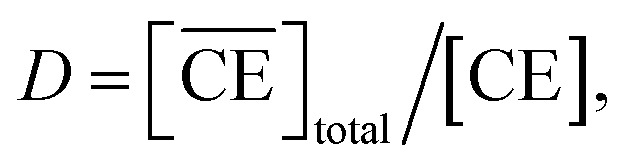
where the *D* value has been reported to be 199.5 in toluene/water for DCH18C6.^44^ The *D* values of the *cis-syn-cis* DCH18C6 isomer are 33, 43, and 180 in 1-octanol/1.00, 3.07, and 6.17 M nitric acid, respectively (for the *cis-anti-cis* isomer, the corresponding values are 48, 54, and 110).^43^ In the present system, it was assumed that the *D* value seems to be of the same order as in previous studies, and thus, set as 1 × 10^2^.

Eqn (8) is rewritten as follows:11
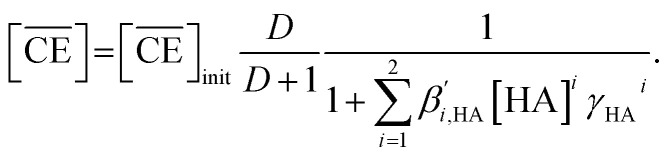


Combining eqn (6) and (11) gives:12
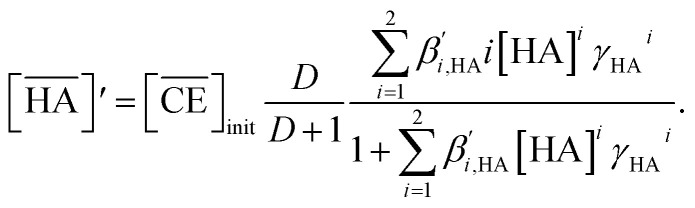


The application of eqn (12) to Fig. 2(a) gives *β*′_1,HNO_3__, and *β*′_2,HNO_3__ values of 0.05 and 0.11, respectively. The latter value is consistent with the value (0.14) reported by Dietz *et al.* for the *cis-syn-cis* DCH18C6 isomer in 1-octanol.^43^ In contrast, the *β*′_1,HNO_3__ value is significantly lower than that of *β*′_2,HNO_3__, and the literature value of 0.54.^43^ A two-fold larger *β*′_2,HNO_3__ value compared to *β*′_1,HNO3_ indicates that HNO_3_ was mainly extracted by DCH18C6 as a 2 : 1 complex of HNO_3_ : DCH18C6 in the present system. Moreover, this result demonstrates that the HFC mixed solvents have the characteristics of a low extraction ability for nitric acid.

In contrast, the corrected concentration of H_2_PFTOUD in the organic phase (
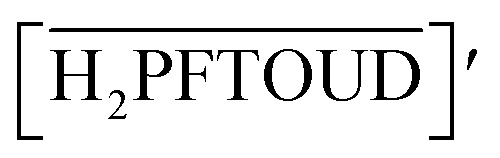
) as a function of the aqueous H_2_PFTOUD concentration ([H_2_PFTOUD]) showed that H_2_PFTOUD was highly extracted into the organic phase in contrast to nitric acid (Fig. 2(b)). To determine the nature of the extracted complex of DCH18C6 and H_2_PFTOUD, the plots in Fig. 2(b) were fitted to a series of equations (eqn (2)–(12)) with the assumption that the conditions were the same as those for nitric acid extraction (HA = H_2_PFTOUD and 
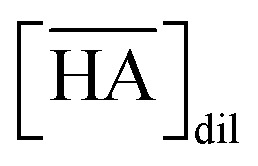
 is shown in Fig. S3†). In the case of H_2_PFTOUD, there is no information concerning the activity coefficients in both the organic and aqueous phases. Thus, the mean activity coefficient (*γ*_H_2_PFTOUD_) was calculated using the Davies equation as follows:13
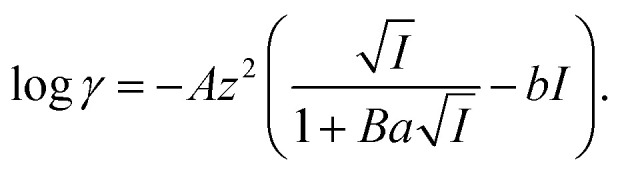


Although H_2_PFTOUD is a dibasic acid based on two carboxyl groups in one molecule, it works as a monobasic acid in Sr(ii) extraction, as described below. Therefore, H_2_PFTOUD was regarded as a monobasic acid in our calculations of the ionic strength in eqn (13). The application of eqn (11) to Fig. 2(b) gives *β*′_1,H_2_PFTOUD_, and *β*′_2,H_2_PFTOUD_ values of 0.87 and 3.5 × 10^2^, respectively. These values are much higher than those obtained from the HNO_3_ system, especially for *β*′_2,H_2_PFTOUD_. The magnitude of *β*′_2,H_2_PFTOUD_ demonstrates that H_2_PFTOUD was well extracted by DCH18C6 into the HFC mixed solvents and was dominated by the formation of a 2 : 1 complex of H_2_PFTOUD : DCH18C6.

The H_2_PFTOUD concentration in the organic phase was >2-fold higher than that of nitric acid at a low acid concentration in the aqueous phase. At sufficiently high aqueous acidities (∼6 M and >0.1 M of nitric acid and H_2_PFTOUD, respectively), the extraction of both nitric acid and H_2_PFTOUD eventually reach a ratio corresponding to 
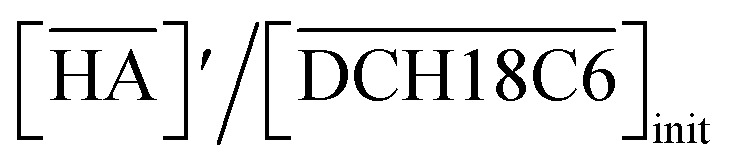
 (HA = HNO_3_ or H_2_PFTOUD) of ∼2 : 1. This result clearly indicates that both acids were mainly extracted as a 2 : 1 complex of acid and DCH18C6 from the aqueous media into the HFC mixed solvents, which is the same as the results obtained upon comparing the conditional extraction constants in each acid.

### Sr(ii) extraction under aqueous nitric acid conditions

**Fig. 3:**
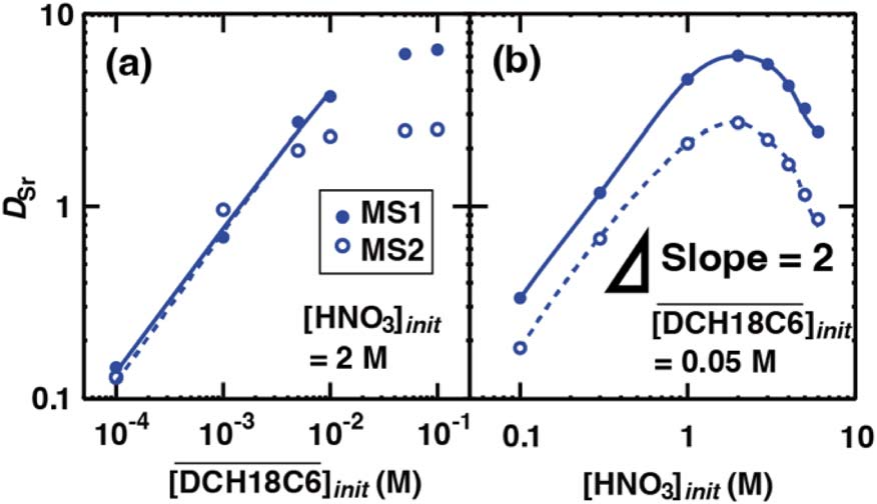
Dependence of *D*_Sr_ on the concentration of DCH18C6 (a) and nitric acid (b) under aqueous nitric acid conditions ([Sr^2+^]_init_ = 0.1 mM). The solid lines and broken lines are fitting curves based on the extraction mechanism described by eqn (17), (18), (20), (27), and (29) in text.

We first investigated the effect of the extractant and acid concentrations on Sr(ii) extraction under aqueous nitric acid conditions. Fig. 3(a) shows the change in *D*_Sr_ for an initial DCH18C6 concentration in the HFC mixed solvents at [HNO_3_]_init_ = 2.0 M (Table S2†). The DCH18C6 concentration dependency exhibited a linear slope at low concentrations, which was expected for the extraction of a 1 : *n* complex with Sr(ii) : DCH18C6 without any side reactions or other competing factors. However, it strongly deviated from linearity at higher concentrations. Moreover, this deviation was particularly pronounced for the HFC mixed solvent MS2. In contrast, Fig. 3(b) depicts the changes in *D*_Sr_ for an initial aqueous HNO_3_ concentration at 
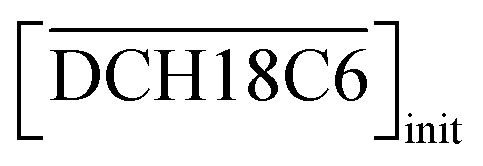
 = 0.05 M (Table S3†). The acid concentration dependency of *D*_Sr_ exhibits a slope of two at low initial concentrations of aqueous HNO_3_, which can be attributed to the neutral complex extraction of Sr(ii). The *D*_Sr_ values change to decrease at 2.0 M. This tendency was in good agreement with the results reported by Gupta *et al.* using conventional diluents such as chloroform and dichloroethane.^26^

To account for these observations, the mechanism of Sr(ii) extraction with DCH18C6 in HFC mixed solvents was considered in detail. The extraction reaction can be assumed to be (A = NO_3_):14
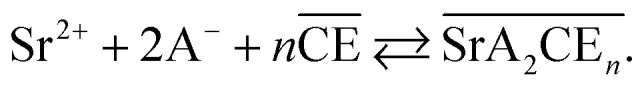


The corresponding extraction equilibrium constant *K*_ex_ is:15
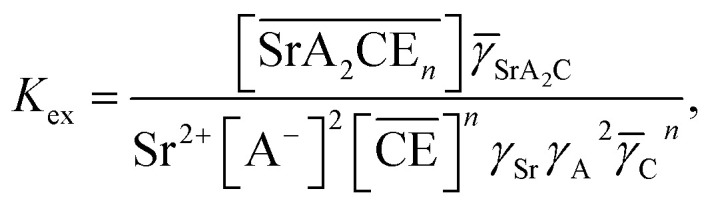
where *γ̄*_SrA_2_C_, *γ*_Sr_, and *γ*_A_ are the organic phase activity coefficients of the 1 : 2 : *n* complex of Sr(ii):nitrate anion:DCH18C6 and the aqueous phase activity coefficients of Sr(ii) and nitrate anions, respectively. If we assume that the ratio between *γ̄*_SrA_2_C_ and *γ̄*_C_ is constant and incorporate them into the conditional extraction equilibrium constant, as shown below:16
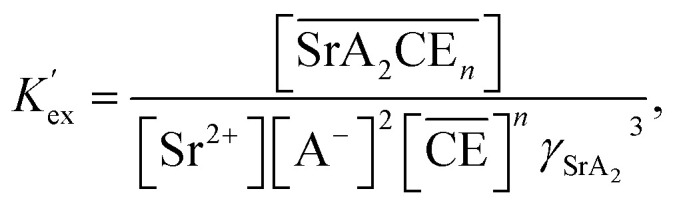
where the mean activity coefficient (*γ*_SrA_2__) was applied to the values calculated using the Davies equation. Simultaneously, the distribution ratio of Sr(ii) yields:17
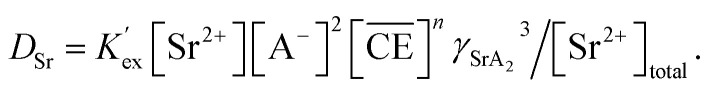


The total mass balance for HA and DCH18C6 is described by modifying eqn (7) and (9), considering both Sr(ii) partitioning (it has been assumed that only SrNO_3_^+^ is present in the aqueous phase under the experimental conditions) and acid dissociation/association:18
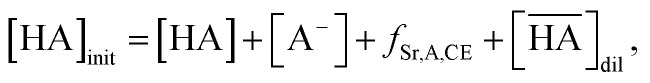
where19
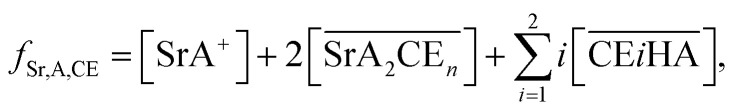
and20
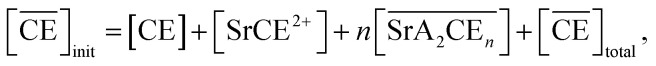
where the relationship between [HA] and [A^−^] can be described as follows (where *α* is the degree of dissociation^45^):

21 ([HA] + [A^−^])*α* = [A^−^]

The formation of SrNO_3_^+^ in the aqueous phase can be represented as follows:22
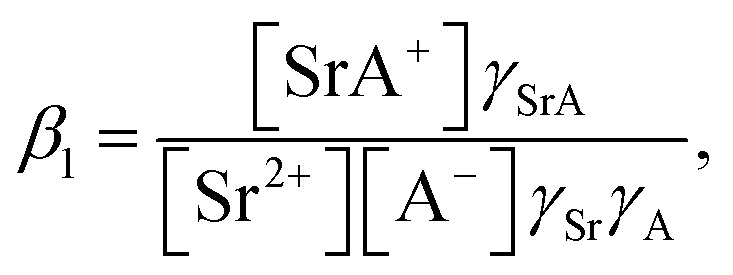
where *β*_1_ is the formation constant of the 1 : 1 complex of Sr(ii) and nitrate anion with a value of 1.15,^46^ and *γ*_SrA_ and *γ*_A_ are the aqueous phase activity coefficients of the complex and nitrate anions, respectively. If we assume that *γ*_H_ = *γ*_SrA_, the relationship can be applied to eqn (22), and its rearrangement gives:23
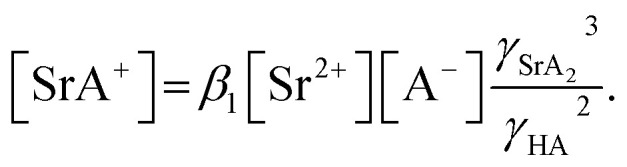


Similarly, the formation of SrCE^2+^ in the aqueous phase can be represented as follows:24
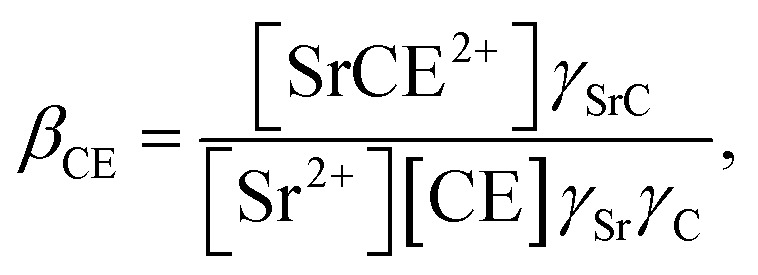
where *β*_CE_, *γ*_SrC_, and *γ*_C_ are the formation constant of the 1 : 1 complex with Sr(ii) : DCH18C6 and the aqueous phase activity coefficients of the complex and DCH18C6, respectively. Assuming that the ratio of the activity coefficients is constant and incorporated into the conditional formation constant, eqn (24) can be rearranged as:

25 [SrCE^2+^] = *β*′_CE_[Sr^2+^][CE].

In the present calculations, the value of *β*′_CE_ was set to 1087, which is the average value obtained from 1-octanol/nitric acid using two DCH18C6 isomers.^43^ Assuming that the partitioning of DCH18C6 can be simplified to 
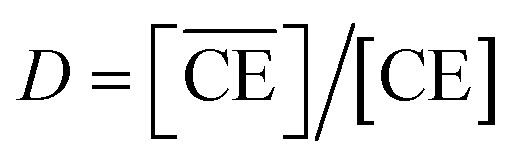
 the DCH18C6 species in the aqueous phase can be written as follows:26
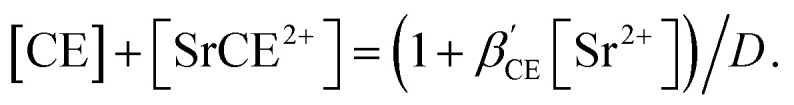


Thus, substituting eqn (6), (16), (25), and (26) into eqn (20) yields:27
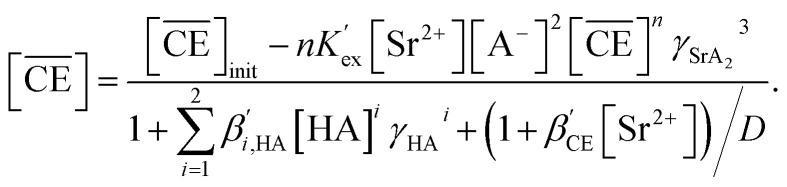


The aqueous concentration of the Sr(ii) species can be represented as follows:

28 [Sr^2+^]_total_ = [Sr^2+^] + [SrA^+^] + [SrCE^2+^]

Substitution of eqn (23), (25), and (26) into eqn (28) gives:29
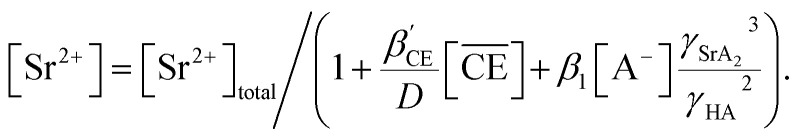


**Fig. 4:**
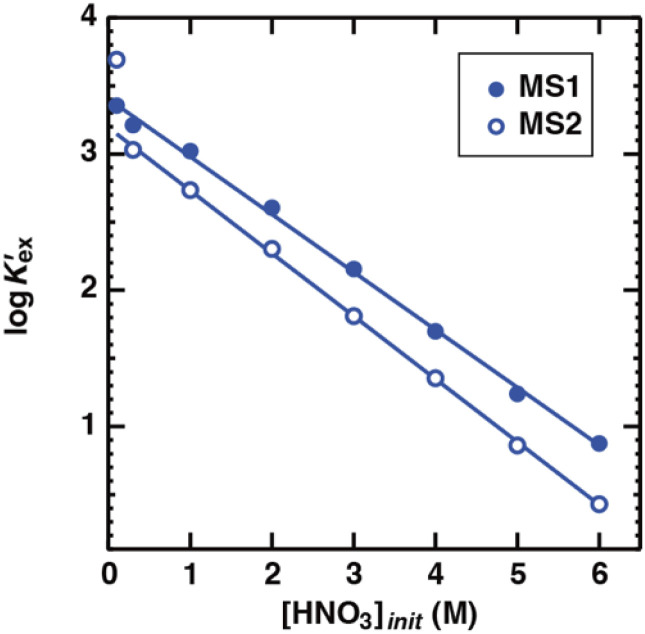
Dependence of log *K*′_ex_ on the concentration of nitric acid under aqueous nitric acid conditions ([Sr^2+^]_init_ = 0.1 mM and 
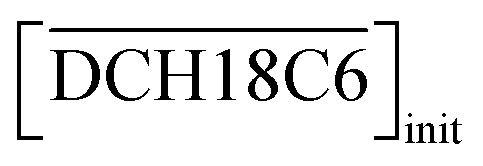
 = 0.05 M).

Eqn (17), (18), (27), and (29) were coupled with eqn (20) and approximated using the least-squares method for the experimentally obtained *D*_Sr_ values in the linear region. In these calculations, the terms that were considered to have a low contribution under the experimental conditions were ignored (specifically, the last two terms in eqn (18), and the last term in the denominator of eqn (27)). The *n* values estimated from the slopes of the fitting curves in Fig. 4(a) were 0.73 (MS1) and 0.77 (MS2), respectively, and the logarithms of the conditional extraction equilibrium constants (log *K*′_ex_) were 2.89 (MS1) and 2.98 (MS2). These values are similar to the value of 2.97 obtained from Sr(ii) extraction by *cis-syn-cis* DCH18C6 isomer to 1-octanol from 3.07 M nitric acid.^43^ The stoichiometry was consistent with that reported in a previous study. Gupta *et al.* reported *n* = 0.75 for Sr(ii) extraction into benzene by DCH18C6 from 2.00 M nitric acid.^26^ These results indicate that both the expressions in the original equations and omissions are reasonable.

Based on the assumption that the composition of the extracted complex was independent of the acid concentration in the aqueous phase, the *D*_Sr_ value was fit using the least squares approximation (Fig. 3(b)). In both HFC mixed solvents, the fitting curves were in good agreement with those obtained from the experiments. The logarithm of *K*′_ex_ calculated by the approximation linearly decreased upon increasing the initial nitric acid concentration (Fig. 4). Although the theoretical background of this linear relationship is still unclear, it is useful for predicting the extraction of Sr(ii) at certain acid concentrations.

**Fig. 5:**
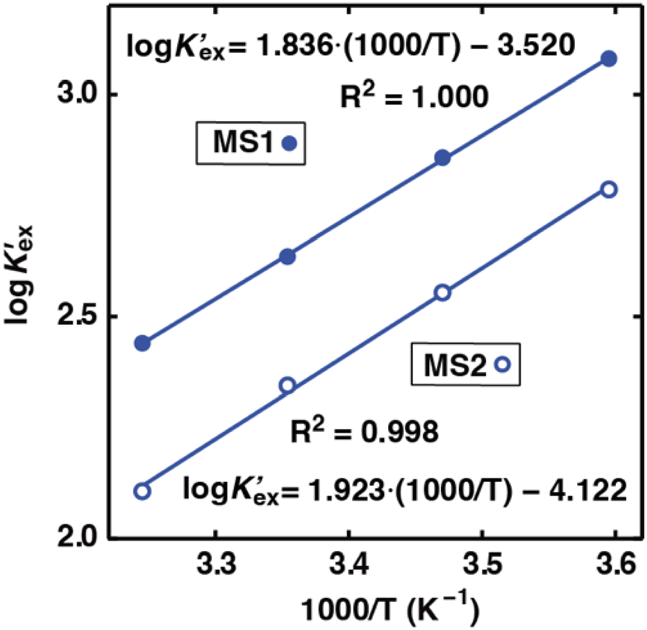
Dependence of *D*_Sr_ on temperature under aqueous nitric acid conditions ([Sr^2+^]_init_ = 0.1 mM, [HNO_3_]_init_ = 2 M, and 
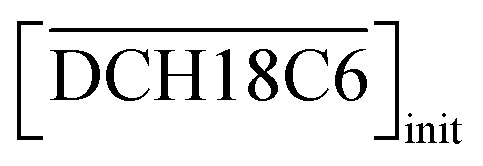
 = 0.05 M).

To gain further insight into Sr(ii) extraction into HFC mixed solvents from nitric acid media, we evaluated the associated thermodynamic parameters. Fig. 5 shows the temperature dependence of *K*′_ex_ under aqueous nitric acid conditions in the temperature range of 5–35 °C (Table S4†). In both MS1 and MS2, the logarithm of *K*′_ex_ linearly decreased with increasing temperature. Typically, the thermodynamic data of the extraction process is obtained using the following relationships: Gibbs energy change (Δ*G*), enthalpy change (Δ*H*), and entropy change (Δ*S*) expressed in eqn (30). The relationship between the extraction equilibrium constant (*K*_ex_) and temperature is described by eqn (31):

30 Δ*G* = Δ*H* − *T*Δ*S* = −2.303*RT* log *K*_ex_,

and31
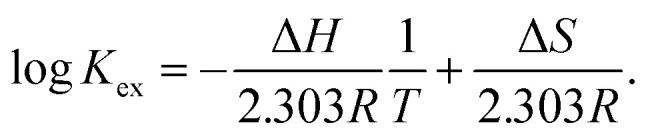


Therefore, by substituting the conditional extraction equilibrium constant calculated from eqn (16) into eqn (31) and fitting the plot of log *K*′_ex_*vs.* 1000/T (Fig. 5), Δ*H* and Δ*S* can be obtained. For MS1, Δ*H* and Δ*S* were determined to be −35.2 kJ mol^−1^ and −67.4 J mol^−1^ K^−1^, respectively. Thus, Δ*G* can be estimated to be −15.1 kJ mol^−1^ at 298 K. Although the values for MS2 are −36.8 kJ mol^−1^, −78.9 J mol^−1^ K^−1^, and −13.3 kJ mol^−1^ (298 K), respectively. These values are considerably negative when compared to those obtained for Sr(ii) extraction using DCH18C6 from 4 M nitric acid into 1-octanol by Kumar *et al.*^39^ (Δ*H* = −19.3 kJ mol^−1^ and Δ*S* = −45.1 J mol^−1^ K^−1^). However, these values are similar to the values obtained using DtBuCH18C6, which is a more hydrophobic neutral complexing agent of an 18C6 derivative (Table 2). These results may be correlated with the extremely low nitric acid solubility in the HFC mixed solvents compared to 1-octanol. In addition, both enthalpy and entropy changes were negative for the HFC mixed solvents. This implies that the interaction between Sr(ii) and crown ether provides negative enthalpy and entropy contributions because the bonding decreases the randomness of the system.^47^

**Table 2:** Thermodynamic parameters for the extraction of Sr(ii) using DCH18C6 and DtBuCH18C6 from a nitric acid solution in HFC mixed solvents or 1-octanol

Extractants	Diluents	[HNO_3_]_init_ (M)	Δ*H* (kJ mol^−1^)	Δ*S* (J mol^−1^ K^−1^)
DCH18C6	MS1	2	−35.2	−67.4
	MS2		−36.8	−78.9
	1-Octanol	4	−19.3 (ref. 39)	−45.1 (ref. 39)
DtBuCH18C6	1-Octanol	1	−39.9 (ref. 48)	−103 (ref. 48)
			−35.3 (ref. 49)	
		3	−28.7 (ref. 48)	−72 (ref. 48)
			−27.1 (ref. 49)	
		4	−25.0 (ref. 39)	−61.9 (ref. 39)

### Sr(ii) extraction under H_2_PFTOUD conditions

For a comparative study of Sr(ii) extraction under aqueous nitric acid conditions, the extraction of Sr(ii) using H_2_PFTOUD as the acid was investigated in terms of the effects of the extractant, Sr(ii), and acid concentrations.

**Fig. 6:**
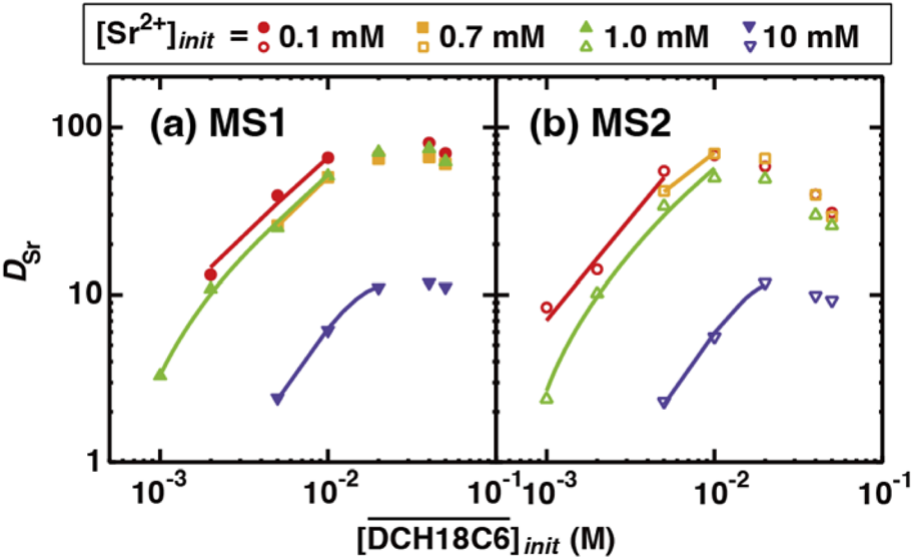
Dependence of *D*_Sr_ on the concentrations of DCH18C6 under H_2_PFTOUD conditions ([Sr^2+^]_init_ and 
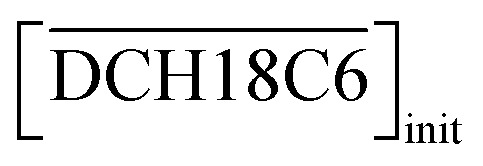
 are shown in the figure and 
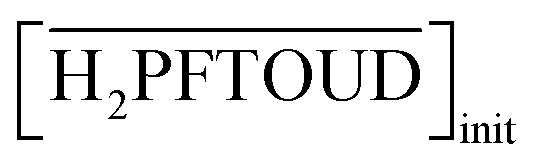
 = 0.02 M). The solid lines are fitting curves based on the extraction mechanism described by eqn (17), (18), (20), (27), (29) and (32) in text.

Fig. 6(a) and (b) show the dependency of *D*_Sr_ on the extractant concentration in the HFC mixed solvents (MS1 and MS2), where the initial H_2_PFTOUD concentration in the organic phase was 0.02 M (Tables S5 and S6†). Both plots show linear slopes at low extractant concentrations, which was expected because of the existence of a 1 : *n* extracted complex with Sr(ii) and DCH18C6 without any side reactions or other competing factors in the HFC mixed solvents. However, the plots deviate markedly from linearity at higher concentrations, which indicates that competing extractions with a lack of Sr(ii), such as the complexation of DCH18C6 with two molecules of H_2_PFTOUD, occurred in this concentration range. The plots of *D*_Sr_ are in good agreement in the concentration range from 0.1 to 1.0 mM of Sr(ii), however, significantly low at 10 mM of Sr(ii) both in MS1 and MS2. This deviation is caused by the concentration competition between Sr(ii) and DCH18C6.

**Fig. 7:**
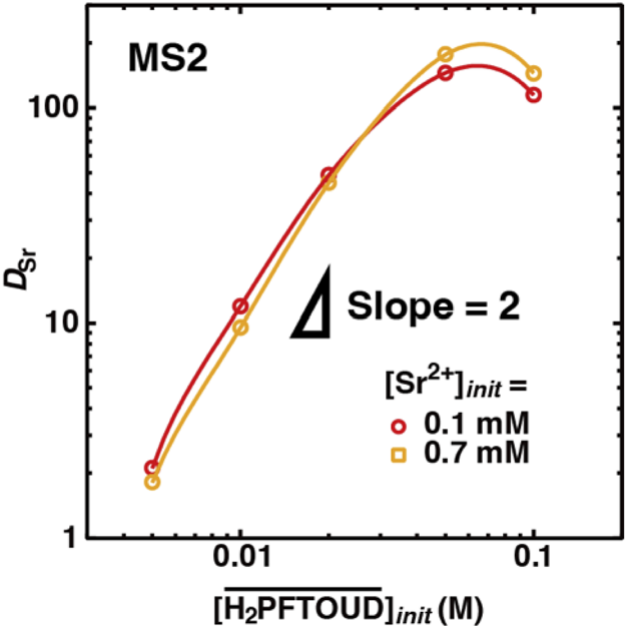
Dependence of *D*_Sr_ on the concentration of H_2_PFTOUD under H_2_PFTOUD conditions ([Sr^2+^]_init_ is shown in the figure and 
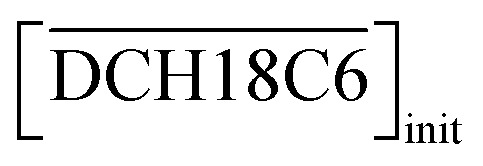
 = 0.02 M). The fitting curves were calculated by the least-squares method based on the extraction mechanism described by eqn (17), (18), (20), (27), (29) and (32) in text.

Fig. 7 shows the dependence of *D*_Sr_ on the initial H_2_PFTOUD concentration in the organic phase, which contains DCH18C6 (initial concentration of 0.02 M) in MS2, from the aqueous phase at two different initial Sr(ii) concentrations (Table S7†). The *D*_Sr_ values increased with increasing H_2_PFTOUD concentration (reaching a maximum at 0.05 M) and the plots exhibited a slope of ∼2 at low concentrations. This result indicates that although H_2_PFTOUD has two carboxyl bases in one molecule, two H_2_PFTOUD molecules exist in one Sr(ii) extracted complex. Based on the concept of neutral complex extraction and the p*K*_a_ value of H_2_PFTOUD (−0.09),^50^ H_2_PFTOUD appears to function as a monovalent acid (*i.e.*, HPFTOUD^−^) in the present system.

Therefore, for a more detailed investigation, the mechanism of Sr(ii) extraction by DCH18C6 and H_2_PFTOUD was considered based on the extraction model used under aqueous nitric acid conditions. Initially, the extraction reaction was assumed to occur *via*eqn (14), where HA is H_2_PFTOUD and acts as a monobasic acid. Simultaneously, the corresponding extraction equilibrium constant (*K*_ex_) can be described using eqn (15). Assuming that the organic phase activity coefficients can be treated in the same manner as that under aqueous nitric conditions, the conditional extraction equilibrium constant (*K*′_ex_) and the distribution ratio can be expressed using eqn (16) and (17), respectively. Subsequently, assuming that the total mass balance (for HA, DCH18C6, and Sr(ii)) and the formula for each chemical species can be described in the same manner as that under aqueous nitric acid conditions, the relationship can also be expressed using eqn (18), (27), and (29). Notably, the degree of dissociation is unknown for H_2_PFTOUD; thus, we assume that the relationship between [HA] and [A^−^] (based on p*K*_a_ = −0.09 and the assumption [H^+^] = [A^−^]) can be described as follows:

32 [HA] = 1.23 × [A^−^]^2^.

Finally, the experimentally obtained *D*_Sr_ values in the region of the linear relationship with DCH18C6 (Fig. 6(a) and (b)) or H_2_PFTOUD (Fig. 7) were fitted using eqn (20) and combined with eqn (17), (18), (27), (29) and (32) using the least squares approximation. The omitted terms were the same as those under aqueous nitric acid conditions, except for the last term in eqn (18), which cannot be omitted because of the high H_2_PFTOUD partitioning into the HFC mixed solvent (Fig. S2†).

The *n* and log *K*′_ex_ values estimated from the fittings in Fig. 6(a) and (b) are summarized in Table 3. In both HFC mixed solvents (MS1 and MS2), the number of DCH18C6 molecules in the Sr(ii) extracted complex (*n*) was almost 1 (0.8–1.2) at the initial Sr(ii) concentration of the aqueous phase between 0.1–1 mM. However, this decreased to 0.6 at 10 mM. Thus, the DCH18C6 stoichiometry of the extracted complex in the former region was essentially the same as that obtained under aqueous nitric acid conditions. By contrast, the log *K*′_ex_ values estimated from the fitting in Fig. 7 (as shown in Fig. S4†), where the *n* values were assumed to be 1.20 and 0.79, were 8.79 and 7.72 for an initial Sr(ii) concentration of 0.1 and 0.7 mM, respectively (Table 3).

**Table 3:** The *n* and log *K*′_ex_ values obtained for Sr(ii) extraction under H_2_PFTOUD conditions at different initial concentrations of strontium

[Sr^2+^]_init_ (mM)	MS1		MS2		Figure No.
	*N*	log *K*′_ex_	*N*	log *K*′_ex_	
0.1	0.97	7.80	1.20	8.52	6
	—	—		8.79^a^	7, S4
0.7	0.95	7.68	0.79	7.52	6
	—	—		7.72^a^	7, S4
1	0.88	7.54	0.93	7.73	6
10	0.60	6.22	0.64	6.32	6

a Average value.

**Fig. 8:**
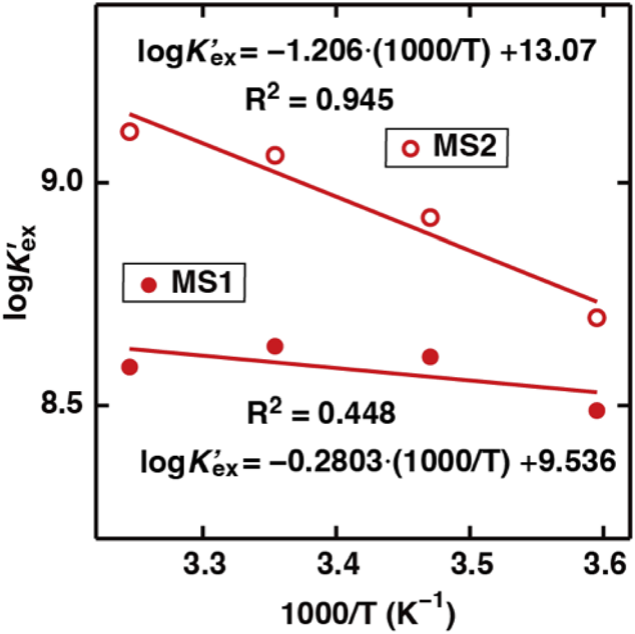
Dependence of *D*_Sr_ on temperature under H_2_PFTOUD conditions ([Sr^2+^]_init_ = 0.1 mM, 
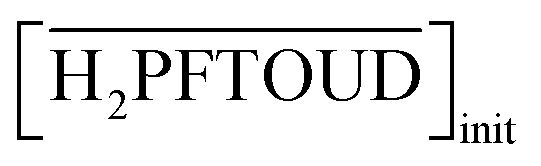
 = 0.02 M, and 
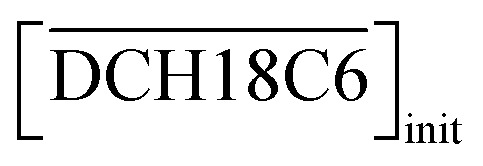
 = 0.02 M).

To obtain the thermodynamic data of the Sr(ii) extraction process using DCH18C6 and H_2_PFTOUD, the *D*_Sr_ values at different temperatures (5–35 °C) were determined (Fig. 8). The relationship between the change in Gibbs energy and changes in enthalpy and entropy is expressed using eqn (31). In addition, the relationship between the extraction equilibrium constant (*K*_ex_) and temperature is described by eqn (30) and (31), where we assume that the extraction equilibrium constant can be substituted for the conditional extraction equilibrium constant. The conditional extraction equilibrium constant (*K*′_ex_) can be calculated using eqn (16), but the mean activity coefficients, such as *γ*_SrA_2__ and *γ*_HA_, cannot be calculated because of the lack of specific data concerning the activity, especially at different temperatures. Therefore, the activity coefficient in this study was applied, as previously calculated, ignoring the temperature parameter. The enthalpy and entropy changes were estimated by applying eqn (31) to line equations represented in Fig. 8, and summarized in Table 4. These results indicate that the extraction process was exothermic and spontaneous. Moreover, the entropy change under H_2_PFTOUD conditions exhibits large positive values in contrast to those under aqueous nitric acid conditions, which implies the existence of an interaction between Sr(ii) and the proton-dissociated H_2_PFTOUD in the extraction process. This “entropy driven” mechanism appears to be due to an increase in the complexation of Sr(ii) with the proton-dissociated H_2_PFTOUD, which increases the randomness of the entropy contribution caused by the dehydration of Sr(ii).

**Table 4:** Thermodynamic parameters for the extraction of Sr(ii) both in the aqueous nitric acid and H_2_PFTOUD conditions

Extraction conditions	Diluents	Δ*H* (kJ mol^−1^)	Δ*S* (J mol^−1^ K^−1^)	Δ*G*^a^ (kJ mol^−1^)
H_2_PFTOUD	MS1	−5.37	182.6	−15.1
	MS2	−23.1	250.2	−13.3
Aqueous nitric acid	MS1	−35.2	−67.4	−67.4
	MS2	−36.8	−78.9	−78.9

a Evaluated at 298 K.

**Fig. 9:**
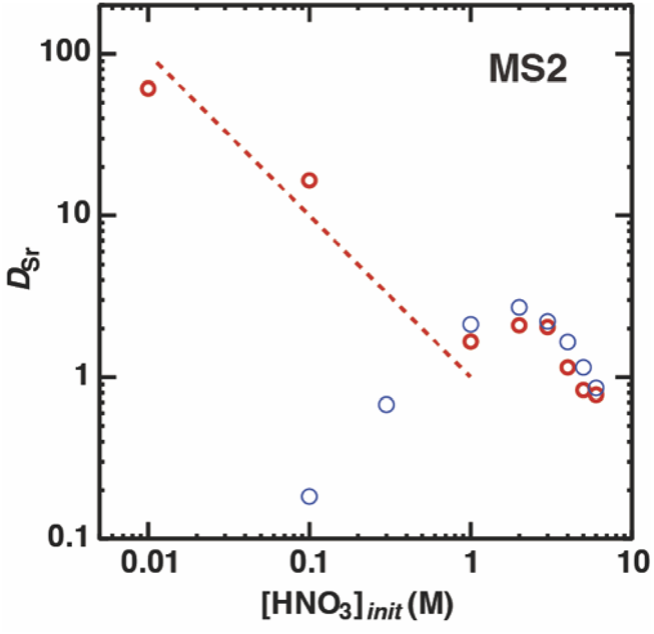
Dependence of *D*_Sr_ on the concentration of nitric acid under H_2_PFTOUD conditions (red, [Sr^2+^]_init_ = 0.1 mM, 
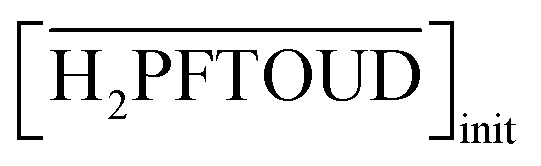
 = 0.02 M, and 
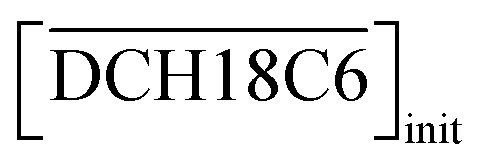
 = 0.02 M); aqueous nitric acid conditions (Fig. 3(a)) were used as the reference (blue).

The previous results indicate that the function of both nitric acid and H_2_PFTOUD was charge neutralization of the extracted Sr(ii) complex during the extraction process. To survey the interactions of the acids during the Sr(ii) extraction process, the extraction behavior of Sr(ii) by DCH18C6 with H_2_PFTOUD was studied by increasing the nitric acid concentration in the aqueous phase. Fig. 9 shows the changes in the *D*_Sr_ value for an initial nitric acid concentration in the aqueous phase of 0.02 M for initial concentrations of DCH18C6 and H_2_PFTOUD in MS2. The *D*_Sr_ values exponentially decrease with increasing HNO_3_ concentration in the range of 0.01 to 1.0 M. At higher concentrations, the values are lower than those obtained with a lack of H_2_PFTOUD (*i.e.*, under aqueous nitric acid conditions, as described in Fig. 3(a)). These results suggest the existence of competitive coordination between proton-dissociated H_2_PFTOUD and nitrate anion with DCH18C6 and Sr(ii) or DCH18C6 during the extraction into the organic phase. At the same time, strong coordination between H_2_PFTOUD and Sr(ii) in the aqueous phase is absent in high nitric acid concentrations.

**Fig. 10:**
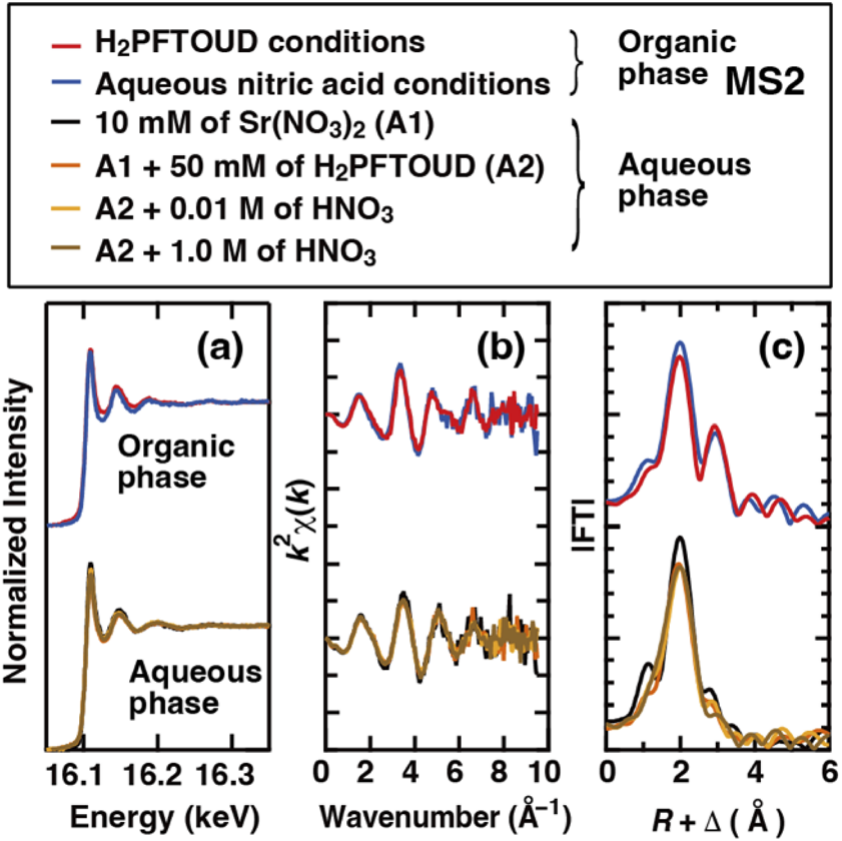
Normalized Sr K-edge XAFS spectra (a), *k*^2^-weighted Sr K-edge EXAFS spectra (b), and Fourier transform data (c) obtained for each phase under H_2_PFTOUD and aqueous nitric acid conditions.

EXAFS measurements provide a powerful way to probe the coordination environment because only the local structure around the absorbing atom contributes to the backscattering of the ejected photoelectron wave. Therefore, XAFS measurements were performed to investigate the environment surrounding Sr(ii) during the extraction process. Fig. 10(a) shows the XAFS spectra of the Sr(ii) species in the organic phases (under H_2_PFTOUDA and nitric acid solution conditions) and in the initial aqueous phases (the strontium nitrate solution: A1, the solution with H_2_PFTOUD added to A1 : A2, and the solution containing different concentrations of nitric acid in A2). The *k*^2^-weighted EXAFS data are shown in Fig. 10(b). Although no clear shift was observed between the EXAFS oscillations of the organic and aqueous phases, their Fourier transformations (Fig. 10(c), without phase-shift-correction) indicate a clear conformational difference for the Sr(ii) species between two phases. In the organic phase, the first and second peaks are located at ∼2 and ∼3 Å, respectively. These peaks were attributed to Sr–O(CE) and Sr–C(CE), respectively, because of the Sr(ii) connected to the crown ethers in the first coordination sphere. This was consistent with the results of Dietz *et al.* using the EXAFS spectra obtained for Sr(NO_3_)_2_(DCH18C6) and Sr(DCH18C6)^2+^ in solution^37^ and Sr(NO_3_)_2_(DtBuCH18C6) in resin.^38^ In contrast, there is a single peak at ∼2 Å in the aqueous phase. Therefore, Sr(ii) mainly exists as a hydrated ion in aqueous solution (the peak is presumably attributed to Sr–O). However, the Sr–O bond lengths of hydrated water and proton-dissociated H_2_PFTOUD appeared to be similar, so they could not be distinguished. Thus, based on the assumption that the proton-dissociated H_2_PFTOUD was coordinated to Sr(ii) in competition with hydration, the solute Sr(ii) species in the aqueous phase should be evaluated. For a more detailed investigation of the extracted complex, a comparison with solid complexes (*e.g.*, Sr(NO_3_)_2_(DCH18C6) and Sr(HPFTOUD)_2_(DCH18C6)) and theoretical fitting using molecular structures will be necessary.

### Effect of γ-ray irradiation on Sr(ii) extraction

The effect of γ-ray irradiation on the organic phase composed of the HFC mixed solvents and DCH18C6 was preliminarily studied. The organic phases were irradiated in glass tubes at γ-ray irradiation doses of 0.5 and 9.8 kGy. In both the MS1 and MS2 solutions, no change in the color of the solution was observed after irradiation at 0.5 kGy. However, a slight amount of white precipitation was observed in the MS1 sample at an irradiation dose of 9.8 kGy (Fig. S5†). These results confirm that the mixtures of HFC-43 directly affect the radiation–resistivity of an HFC mixed solvent and that the *trans*-1,2-dichloroethylene moiety with a double carbon–carbon bond was extremely sensitive to γ-radiation in contrast to heptane. Therefore, MS2, which was composed of HFC-43 and heptane, was used as the diluent in the extraction experiments.

**Fig. 11:**
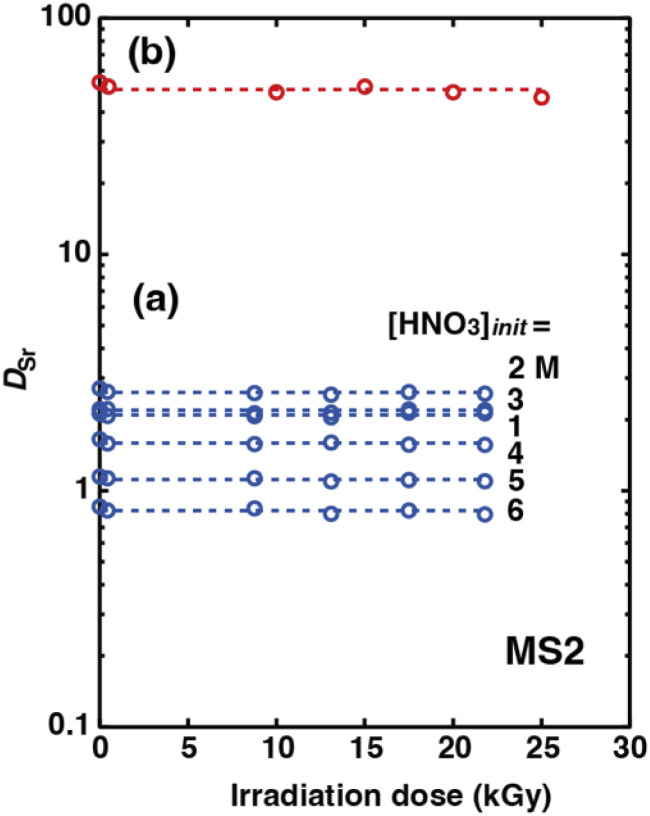
Effects of the irradiation dose on *D*_Sr_ under aqueous nitric acid conditions (a, [Sr^2+^]_init_ = 0.1 mM, 
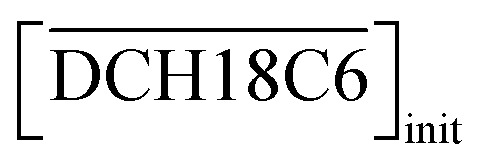
 = 0.05 M) and H_2_PFTOUD conditions (b, [Sr^2+^]_init_ = 0.1 mM, 
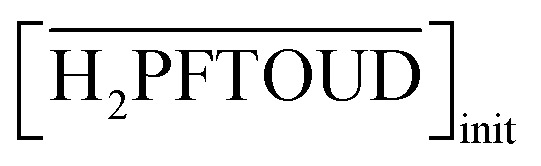
 = 0.02 M, and 
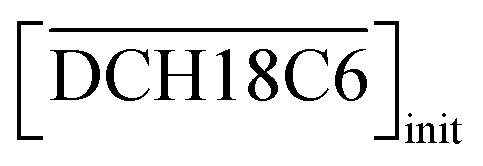
 = 0.02 M). The dotted lines show the average *D*_Sr_ values under each condition.

The organic phases were irradiated in glass tubes with γ-ray irradiation doses of 0.4, 9, 13, 18, and 22 kGy under aqueous nitric acid conditions and 0.5, 10, 15, 20, and 25 kGy under H_2_PFTOUD conditions prior to the extraction experiments. In the case of MS2, no precipitation and no change in the color of the solution were observed even for the sample subjected to an irradiation dose of 25 kGy (Fig. S6† shows the case under H_2_PFTOUD conditions). Fig. 11(a) shows the dependency of *D*_Sr_ on the irradiation dose at each initial nitric acid concentration in the aqueous phase (Table S10†). The *D*_Sr_ values at each acid concentration were constant and independent of the irradiation dose. This result indicates that heptane or HFC-43 takes over the damage caused by the absorbed dose, not DCH18C6. Moreover, the radiolytic decomposition products of heptane and HFC-43 seem to be soluble and have no effect on the Sr(ii) extraction process. Similarly, Fig. 11(b) shows the dependency of *D*_Sr_ on the irradiation dose under H_2_PFTOUD conditions (Table S11†). The trend observed for *D*_Sr_ was consistent with the results obtained under aqueous nitric acid conditions. These results demonstrate that the irradiation damage on not only the extractant, but also the acids, DCH18C6 and H_2_PFTOUD, was quite small and can be considered negligible. For practical use, further investigation of heavier γ-irradiation is required.

## Conclusions

The present study overcomes the solubility problem in fluorinated solvents of crown ethers with high extraction ability of Sr(ii) and confirms a good extraction system by the combination of DCH18C6 and two HFC mixed solvents (30/60 (w/w)% *trans*-1,2-dichloroethylene/HFC-43 and 5/95 (w/w)% heptane/HFC-43). Perfuruoro-3-6-9-trioxaundecane-1,11-dioic acid (H_2_PFTOUD) efficiently promoted the Sr(ii) extraction process and showed a good distribution ratio when compared with nitric acid (the maximum *D*_Sr_ values observed for the former are ∼180, and >10 times larger than the latter) implying that its fluorophilic property plays an important role in the extraction of Sr(ii) into HFC mixed solvents. The composition of extracted complexes was estimated using slope analysis as an Sr(ii):acid anion:DCH18C6 ratio of ∼1 : 2 : 1. EXAFS measurement indicated that the first coordination sphere of the extracted complex is occupied by a DCH18C6 molecule, regardless of the acid used. This work provides a series of thermodynamic data that contribute to a better understanding of the extraction process of Sr(ii) by crown ether in HFC mixed solvents. The results indicate that the complexation of proton-dissociated H_2_PFTOUD, Sr(ii), and DCH18C6 is accompanied by dehydration during extraction process. This mechanism, so-called entropy driven mechanism, contributes stabilization of the Sr(ii) extracted species in HFC media. Though verification on a larger scale is needed for practical application, the results obtained from the present study will further help in the understanding of entropy driven extraction mechanism, and hence in the improved design of systems for metal extraction.

## Author contributions

Kenji Shirasaki: conceptualization, data curation, formal analysis, investigation, methodology, resources, software, supervision, visualization, writing – original draft, writing – review & editing. Mitsuie Nagai: data curation, resources. Masahiko Nakase: data curation, formal analysis, software, writing – review & editing. Chihiro Tabata: validation, writing – review & editing. Ayaki Sunaga: validation, writing – review & editing. Tsuyoshi Yaita: data curation, formal analysis, resources, Tomoo Yamamura: conceptualization, funding acquisition, investigation, methodology, resources, supervision, Writing – review & editing.

## Conflicts of interest

There are no conflicts to declare.

## Supplementary Material
